# The evolution of transposable elements in natural populations of self-fertilizing *Arabidopsis thaliana *and its outcrossing relative *Arabidopsis lyrata*

**DOI:** 10.1186/1471-2148-10-10

**Published:** 2010-01-12

**Authors:** Steven Lockton, Brandon S Gaut

**Affiliations:** 1 Department of Ecology and Evolutionary Biology, University of California, Irvine, USA

## Abstract

**Background:**

Transposable Elements (TEs) make up the majority of plant genomes, and thus understanding TE evolutionary dynamics is key to understanding plant genome evolution. Plant reproductive systems are diverse and mating type variation is one factor among many hypothesized to influence TE evolutionary dynamics. Here, we collected a large TE-display data set in self-fertilizing *Arabidopsis thaliana*, and compared it to data gathered in outcrossing *Arabidopsis lyrata*. We analyzed seven TE families in four natural populations of each species to tease apart the effects of mating system, demography, transposition, and selection in determining patterns of TE diversity.

**Results:**

Measures of TE band differentiation were largely consistent across TE families. However, patterns of diversity in *A. thaliana Ac *elements differed significantly from that other TEs, perhaps signaling a lack of recent transposition. Across TE families, we estimated higher allele frequencies and lower selection coefficients on *A. thaliana *TE insertions relative to *A. lyrat*a TE insertions.

**Conclusions:**

The differences in TE distributions between the two *Arabidopsis *species represents a synthesis of evolutionary forces that include the transposition dynamics of individual TE families and the demographic histories of populations. There are also species-specific differences that could be attributed to the effects of mating system, including higher overall allele frequencies in the selfing lineage and a greater proportion of among population TE diversity in the outcrossing lineage.

## Background

Transposable elements (TEs) are prevalent in plant genomes [1] and ubiquitous among eukaryotes [2]. Although TEs comprise most of an average plant genome [3], their content varies markedly among populations [4,5] and species [6,7]. For example, TEs make up ~70% of the maize genome [8] but just 10% of the *Arabidopsis thaliana *genome [9]. Moreover, TEs can accrue rapidly after polyploid and hybrid speciation events [10,11]. These observations raise questions about the evolutionary forces that govern the distribution of TEs within plant genomes.

Population genetics has the potential to illuminate these forces, but our understanding of the population genetics of TEs has been based primarily on studies of *Drosophila melanogaster*. These studies have revealed that there are far fewer TE insertions in the *D. melanogaster *genome than possible insertion sites [12,13] and that insertions tend to be at low population frequencies [12-14]. Both observations suggest that the spread of TEs is countered by natural selection [15-18]. However, the precise nature of selection against insertions is unclear. Some insertions may disrupt gene products or gene expression [19]. Purifying selection against these deleterious insertions could be the driving force that governs observed TE distributions [15,19-23]. Another possibility is that TEs facilitate deleterious chromosome rearrangements through non-homologous (or ectopic) recombination [18,24-28].

The mating system of host species is likely to be an important factor that shapes TE dynamics [27,29,30]. For example, in highly homozygous selfing species most TEs have a paired homologous allelic partner, which reduces the probability of an ectopic recombination event [27,29,30]. If selection against TEs is primarily mediated by these ectopic events, then selfing species are predicted to have less efficacious selection and *higher *TE copy numbers than outcrossing species. Conversely, the deleterious effects of recessive TE insertions are expected to be stronger in a homozygous selfer, which may result in more efficacious selection and *fewer *TEs in selfers [20,29,30]. Thus, the effect of breeding system is difficult to predict precisely, but simulations of TE population dynamics provide evidence to support the possibility that both ectopic recombination and deleterious insertions will lead to differences in TE accumulation between selfers and outcrossers [29,30].

Mating system influences the efficacy of selection against TEs in at least two other ways: First, the effective population size (*N*_*e*_) in a selfing species is expected to be half that of an otherwise identical outcrosser [31,32]. Population size has a direct effect on the efficacy of selection, because efficacy is reflected in the compound parameter *N*_*e*_*s*, where *s *is the strength of selection. It is thus not surprising that empirical studies suggest that shifts in *N*_*e *_over time influence the number and frequency of TEs [5,33]. Second, inbreeding reduces the effective recombination rate, which may lead to the accumulation of weakly deleterious TE insertions [34] via Hill-Robertson effects [35]. Observations that TEs accumulate on non-recombining sex chromosomes support this conjecture [36,37].

Despite predictions that TE population dynamics may differ markedly between selfing and outcrossing species, comparative data are quite rare. Recently, however, Dolgin *et al*. [38] documented that population frequencies of *Tc1*-like insertions are higher in selfing *Caenorhabditis elegans *than in outcrossing *C. remanei*. This pattern of diversity suggests less efficacious selection against insertions in the selfing species; indeed, Dolgin *et al*. [38] tentatively conclude that *Tc-1 *element insertions are effectively selectively neutral in *C. elegans*.

Plants are particularly well suited for inter-species comparisons of TE population dynamics because of broad diversity in mating systems. Studies of selfing and cultivated *Lypersicon *species have generally shown differences in TE complement that are consistent with less efficacious selection against insertions in selfing species. For example, the *Lyt1 *element family has higher copy numbers in the selfing members of the genus [27,39], and *copia*-like insertions are generally found at higher population frequencies in selfers [40]. In perhaps the best known study TE diversity between plant species with contrasting mating systems [41], Wright et al. [42] compared population diversity of *Ac*-like elements between selfing *Arabidopsis thaliana *and outcrossing *A. lyrata*. *Ac*-like insertions were slightly more numerous in *A. thaliana *but segregated at significantly lower frequencies in *A. lyrata*, consistent again with reduced efficacy of natural selection against insertions in the selfing lineage.

Although the limited data gathered to date suggests that selection against TEs is less efficacious in selfing lineages, it is difficult to determine whether extant patterns of TE diversity are due to the effects of selection or complicated by other factors that may differ between species, such as demographic history and transposition dynamics [42]. How might one discriminate among these factors? One approach is to increase sampling to multiple TE families and multiple populations. If patterns of TE diversity vary across element families, transposition dynamics may play a major role in explaining differences between species like those observed for *Ac*-like elements [42]. In contrast, if diversity patterns are consistent across TE families, forces that affect whole genomes (such as demography and breeding system) may be the primary determinants of TE diversity. Here we extend the study of Wright et al. [42] to contrast TE population genetics between *A. thaliana *and *A. lyrata*, generating polymorphism profiles from four populations of *A. thaliana *representing seven TE families. We compare these *A. thaliana *data to data gathered from four populations of the outcrossing congener *A. lyrata *[5]. By contrasting TE frequencies and patterns across species, populations, and TE families, we gain insight into the relative roles of transposition, demography, and breeding system in shaping TE diversity.

## Methods

We sampled four populations of *A. thaliana *with seed obtained from The *Arabidopsis *Information Resource (TAIR [43]). The sample included 12 individuals from Ascot, U.K. (TAIR seed stock numbers CS22220-CS22235), 12 from Anholt, Germany (CS22313-CS22324), 12 from Knox, Indiana, USA (CS22401-CS22412), and 11 individuals from Cold Spring Harbor, New York (CS22419-CS22430). Plants were grown in a growth chamber for eight weeks, and DNA was extracted from leaf material. Our TE display procedure followed [5], including the extensive technical replication, to produce *A. thaliana *TE polymorphism data for *Ac*-like III (henceforth "*Ac*"); *Helitron Basho *TEs ("*Basho*"); CACTA; *Gypsy*-like ("*Gypsy*"); LINE-like ("LINE"); SINE-like I ("SINE"); and *Tourist*-like MITE ("MITE") elements. These TEs represent three RNA-mediated class I retrotransposons (LINE, SINE and *Gypsy*) and four class II DNA transposons (*Ac, Basho*, MITE and CACTA). The primers used to generate *Ac *TE-display data were identical to those used by Wright *et al*. [42]. We also utilized the TE display data from [5], encompassing 44 individuals from four natural *A. lyrata *populations: 11 individuals from Plech, Germany, 12 from Karhumäki, Russia, 12 from North America, and nine from Stubbsand, Sweden.

### Molecular Analysis of Variance

To measure levels of population differentiation in our *A. thaliana *sample, we performed a Molecular Analysis of Variance (AMOVA) [44]. We focused on Φ_*PT*_, a statistic analogous to *F*_*ST *_that measures genetic differentiation among populations. For our analyses, we used TE-display bands as genetic markers, and thus Φ_*PT *_became a measure of TE display band differentiation. Our analyses were performed with two different packages: GenAlEx 6 [45] was used to compare Φ_*PT *_between populations and the R package ade4 [46] was used to calculate Φ_*PT *_among all populations.

### Allele frequencies and copy numbers

We compared our *A. thaliana *TE-display data to *A. lyrata *data by estimating TE allele frequencies and copy numbers in both species. We used estimates of the inbreeding coefficient (*F*) to estimate TE allele frequencies from dominant TE display data. For each *A. lyrata *population, *F *was estimated independently using (SNP) data in 77 loci [47] by , where  is the estimated inbreeding coefficient,  is the average observed heterozygosity per locus [48], and  is the average expected heterozygosity, under random mating, calculated by

where *p*_*i *_is the frequency of the *i*th of *k *alleles, summed over the *l*th of *m *SNP loci [48].

Ross-Ibarra et al. [47] sampled the same German, Russian, and Swedish populations, and *F *was estimated directly for these populations. However, they sampled two North American populations (Ontario, Canada and Indiana, USA) that were combined to yield our North American sample. We thus average  between these two populations to procure an estimate of *F *for our North American sample. To estimate *F *in *A. thaliana*, we assumed the proportion of selfing (*S*) in *A. thaliana *was 0.99 [49] and estimated *F *= 0.98 from the relation *F *= *S*/(2 - *S*) [50].

Given estimates of *F*, we estimated *p*_*TE*_, the TE allele frequency, using(1)

[51], where *z *is the observed frequency of the null TE display band (*i.e*., 1 - the population frequency of the dominant TE band), *q *is the estimated null TE allele frequency, and *q *= 1 - *p*_*TE*_. We calculated allele frequency estimates both within populations and across entire species' samples.

We calculated *n*_*TE*_, the expected TE copy number of an individual, as:(2)

where *p*_*i *_is TE allele frequency of the *i*th locus, summing over *m *TE loci. *I*_*i *_is an indicator variable, where *I*_*i *_= 1 when a TE band is present, and *I*_*i *_= 0 when a TE band is absent, at the *i*th locus in a given individual. Bands fixed within our sample were included in our calculations of allele frequencies and *n*_*TE*_.

### Estimation of selection coefficients

We used the Maximum Likelihood (ML) approach of Petrov et al. [18], with modifications introduced by Lockton et al. [5], to estimate the population-selection coefficients (*N*_*e*_*s*) from our TE display data. Lockton et al. [5] modified the method to correct for ascertainment biases inherent in TE-display data and also to employ . In this method, *N*_*e*_*s *is compound parameter, but following Petrov et al. [18] we assume values for *N*_*e *_based on nucleotide polymorphism data. We used the point estimates of *N*_*e *_inferred from demographic modeling of the same four *A. lyrata *populations - i.e., Germany *N*_*e *_= 136,000; North America *N*_*e *_= 11,000; Russia *N*_*e *_= 12,000; and Sweden *N*_*e *_= 12,000 (Ross-Ibarra et al., 2008). Species-wide *A. lyrata N*_*e *_was calculated to be 250,000 by using estimates of *θ *from SNP data [47], and assuming a mutation rate (*μ*) of 1.5 × 10^-8 ^[52]. For *A. thaliana*, we also used estimates of *θ *from SNP diversity data [53] to estimate *N*_*e*_, assuming *μ *= 1.5 × 10^-8 ^[52]. Species-wide *N*_*e *_was estimated to be 125,000; the UK population was 98,500; Germany, 83,000; and both New York and Indiana was 71,000. However, results did not differ qualitatively when *N*_*e *_was assumed to be 125,000 in each *A. thaliana *population (data not shown).

## Results & Discussion

### TE Display Bands

We identified 267 TE display bands in *A. thaliana *across seven TE families. To compare, in *A. lyrata*, 274 bands were amplified in six TE families [5]. Of the six TE families shared between species (*Ac*, CACTA, *Gypsy*, LINE, MITE, and SINE) there were more TE bands in outcrossing *A. lyrata *(*n *= 274) than in the self-fertilizing *A. thaliana *(*n *= 210). A sample of the bands amplified using TE family-specific primers were cloned, sequenced, and identified: 95% (20/21) of the *A. thaliana *bands were successfully identified as TEs belonging to their respective families (data not shown). The single unidentified *A. thaliana Ac *band showed strong sequence similarity to an "unknown protein" (BLASTn e-value: 5e-102) in the *A. thaliana *genome sequence.

We readily identified 57 *Basho *bands in *A. thaliana*, but few strong bands in *A. lyrata*. The putative *Basho *bands that were amplified, cloned, and sequenced from *A. lyrata *could not be identified in TE databases using BLAST. Because of the uncertainty of the *A. lyrata Basho *data, they were not included in additional analyses. These empirical results are consistent with previous studies suggesting that some *Basho *subfamilies may be absent from *A. lyrata *[54].

### Molecular Analysis of Variance

**Figure 1:**
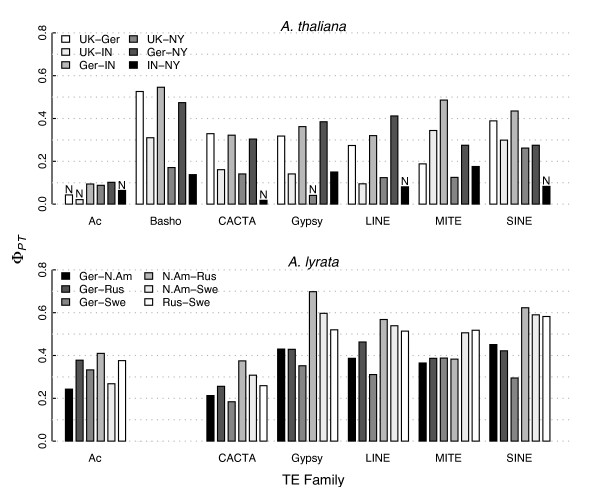
**AMOVA Φ_*PT *_per TE family in each population pairwise comparison in both species. *A. lyrata *data from **[5]. *A. thaliana *populations: UK, Ascot, United Kingdom; Ger, Anholt, Germany; IN, Knox, Indiana, USA; NY, Long Island, New York, USA. *A. lyrata *populations: Ger, Plech, Germany; N.Am, North America; Rus, Russia; Swe, Sweden. "N"s indicate comparisons that show no significant population differentiation (Φ_*PT *_= 0, *p *> 0.05).

We utilized an AMOVA to examine *A. thaliana *band differentiation between populations for each TE family (Fig. 1). Overall, *A. thaliana *tends to have lower levels of Φ_*PT *_between populations relative to *A. lyrata *[5]. Higher Φ_*PT *_values for *A. lyrata *are consistent with its more disjunct distribution [55,56], its high nucleotide diversity [47], and its relatively large and stable populations [57].

Nonetheless, Φ_*PT *_values between *A. thaliana *populations are typically significantly > 0, as might be expected of a species that is increasingly recognized as having considerable population structure [58-60]. The Φ_*PT *_values mirror geographic distances in some cases. For example, the lowest Φ_*PT *_tended to be between the populations geographically closest to each other (Fig. 1; Indiana, USA, and Long Island, NY, USA; non-significant in 4/7 TE families), which may reflect low genetic structure among human-commensal North American *A. thaliana *populations [53,61]. It is striking, however, that for most TE families we also observe low Φ_*PT *_between the UK and the US population samples (Fig. 1). Indeed, our UK sample appeared to have less TE band differentiation *vs*. each of the U.S. samples than UK *vs*. Germany (Fig. 1), even though the two European populations are closer geographically. The striking difference between the UK and German populations could reflect, in part, patterns of migration across Europe [60] and particularly the possibility of admixture in Central Europe from separate glacial refugia [58,62]. To our knowledge genetic similarity between US and UK populations has not been noted previously.

**Figure 2:**
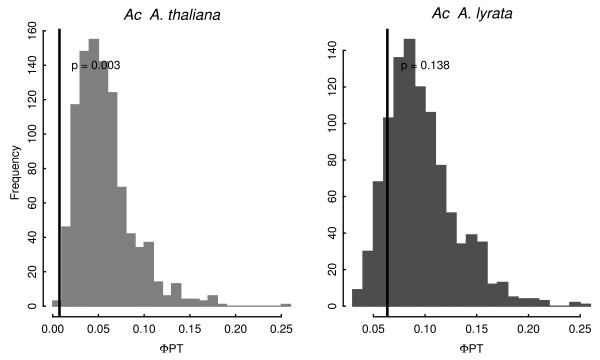
***Ac *AMOVA Φ_*PT *_compared to bootstrap replicates subsampled from all TE family TE display bands**.

Arguably the most striking aspect of pairwise Φ_*PT *_values is relatively low values for *A. thaliana Ac *elements (Fig. 1), suggesting that *Ac *population dynamics differ from those of the other TEs surveyed. To test this idea more formally, we estimated total Φ_*PT *_values among all populations for each TE family, and then compared the observed values to Φ_*PT *_from bootstrapped replicates. The bootstrap samples were based, first, on combining bands across TEs, under the null hypothesis that all TE families are representative of a homogeneous evolutionary process. Then, for each TE family, bootstrap replicates mimicked the observed number of bands from each population and, finally, Φ_*PT *_was calculated for each replicate. From this exercise, it is clear that Φ_*PT *_from *A. thaliana Ac *is much lower than expected under the null hypothesis (p = 0.003; Fig. 2). In contrast, data from *A. lyrata Ac *elements did not reject the null hypothesis of homogeneity (Fig. 2), nor did TE data from any other TE family in either species after multiple-test correction (data not shown). Thus, population genetic information does vary among TE families, with *A. thaliana Ac *an obvious outlier.

**Figure 3:**
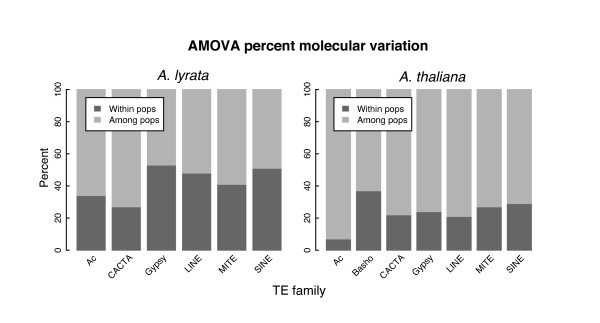
**AMOVA percent molecular variation within and among populations ("pops")**.

We also estimated variance components for each TE family in both species using AMOVA (Fig. 3). If breeding system has an appreciable effect on TE diversity, a selfer should exhibit less TE band diversity within each population than among populations, because inbreeding leads to populations with low genetic diversity [63]. Our data are consistent with this prediction: Among-population variation was proportionally higher in *A. thaliana *compared to *A. lyrata *across all TE families (two-tailed sign test, *p *= 0.03; Fig. 3). One must be careful about interpreting these results, however, as differences in sampling could contribute to apparent differences between species. Indeed, our *A. thaliana *TE-display suggests that our within-population variation is a smaller component than found in a previous population study based on combined microsatellite and SNP data [59]. Nonetheless, the partitioning of variation is consistent across TE families, and does suggest some genome-wide effect of species with regard to the partitioning of TE variation. In addition, variance components graphically demonstrate that *A. thaliana Ac *elements differ from other elements with regard to the distribution of diversity (Fig. 3).

### TE insertion frequencies and the strength of selection

**Figure 4:**
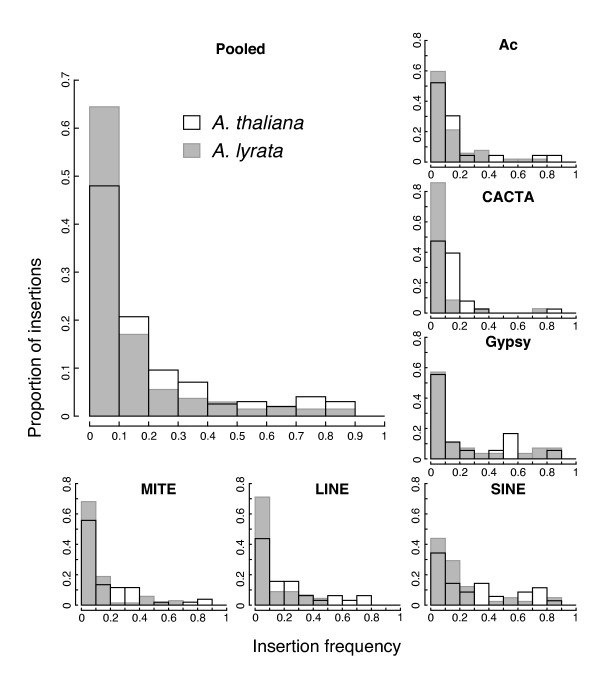
**Site Frequency Spectra of all pooled TEs ("Pooled"), and each TE family in species-wide samples of *A. thaliana *(black bordered bars) and *A. lyrata *(grey bars with grey borders)**.

AMOVA utilizes TE-display bands, but allele frequencies are often more helpful for evaluating evolutionary dynamics. For TE-display data, a band from an inbred species is more likely to represent a homozygous locus than bands in an outcrossing species. We therefore used independent estimates of the inbreeding coefficient (*F*) to estimate allele frequencies (*p*_*TE*_) from our TE band data (see Methods). This method intrinsically corrects for possibilities that *A. lyrata *may not always be obligately outcrossing (e.g., [64]) but does assume a constant rate of selfing in *A. thaliana*. With *p*_*TE *_estimates, we can examine site frequency spectra (SFS), which form the basis for inferring the strength of selection [65,66]. We combined data across populations to construct species-wide samples. Species-wide, and across all TE families, the *A. lyrata *median *p*_*TE *_was 0.061, but the *A. thaliana *median *p*_*TE *_of 0.125 was substantially higher (Table 1; Wilcoxon rank sum test, *p *= 1.28 × 10^-6^). In addition to lower median frequencies, the *A. lyrata *SFS showed a skew towards lower frequency insertions in *A. lyrata *relative to *A. thaliana *(Fig. 4). This skew is evident not only for the SFS pooled among TE families, but also for most individual TEs (Fig. 4). The standard interpretation of a left-skewed SFS is that purifying selection acts on deleterious variants, limiting their population frequencies. Thus, the skew in *A. lyrata *relative to *A. thaliana *is consistent with stronger selection acting on TEs in *A. lyrata*, as concluded by Wright et al. [42] for *Ac *elements alone.

**Table 1:** Median per individual TE allele frequency (*p*_*TE*_)

	*A. lyrata*						*A. thaliana*					
	Plech, Germany	N. America	Russia	Sweden	Mean	Standard Deviation	Anholt, Germany	IN, USA	NY, USA	Ascot, UK	Mean	Standard Deviation
*Ac*	0.221	0.208	0.291	0.31	0.258	0.051	0.192	0.231	0.324	0.221	0.242	0.068
*Basho*	-	-	-	-	-	-	0.166	0.33	0.351	0.305	0.288	0.102
CACTA	0.159	0.235	0.23	0.184	0.202	0.037	0.298	0.224	0.212	0.256	0.248	0.047
*Gypsy*	0.247	0.337	0.315	0.344	0.311	0.044	0.289	0.278	0.286	0.269	0.281	0.005
LINE	0.237	0.2	0.15	0.282	0.217	0.056	0.222	0.339	0.329	0.259	0.287	0.065
MITE	0.133	0.164	0.291	0.309	0.224	0.089	0.136	0.311	0.313	0.245	0.251	0.102
SINE	0.23	0.366	0.274	0.28	0.288	0.057	0.249	0.299	0.342	0.308	0.300	0.046

**Figure 5:**
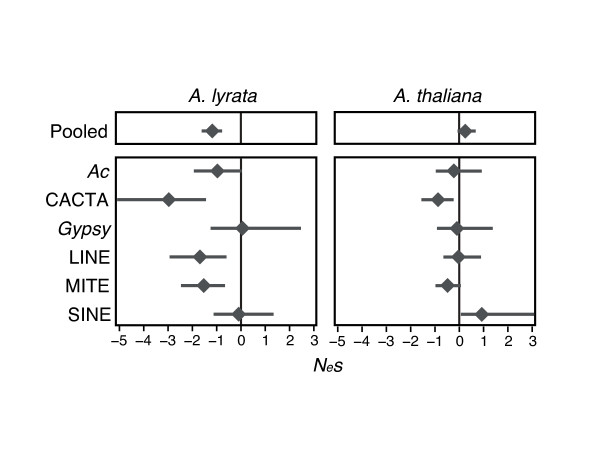
**Maximum Likelihood *N*_*e*_*s *estimates for in species-wide samples of *A. thaliana *and *A. lyrata *in pooled TEs ("Pooled") and each TE family**. Horizontal bars are the 95% Confidence Interval and the diamond represents the ML *N*_*e*_*s *point estimate. The vertical line is *N*_*e*_*s *= 0.

If we assume a transposition-selection equilibrium, the strength and direction of selection can be estimated from the SFS using an ML framework [18]. The ML approach, as implemented here, incorporates information about inbreeding *F *into the model [5]. Applying this approach, *A. thaliana*'s *N*_*e*_*s *estimates for pooled TEs were not significantly different from zero, and individual TEs pooled across populations also yielded *N*_*e*_*s *estimates very close to zero (Fig. 5; SI Table 1). The corresponding estimates were lower in *A. lyrata *[5], and the total sample of TEs yielded an *N*_*e*_*s *point estimate significantly less than zero, at -1.9 (Fig. 5; SI Table 1).

The SFS and *N*_*e*_*s *results are consistent with a species-wide reduction in the strength of selection in the selfing species compared to the outcrossing species. This lends superficial support either to mechanisms of selection (such as ectopic recombination) that are hypothesized to be more prevalent in an outcrosser, or to complicating factors in selfers (such as interference due to linkage or smaller population sizes) that slow selection [18,20,22,26]. However, these 'species-wide' results could also be an artifact of combining samples across populations. Under this line of reasoning, the skewed SFS in *A. lyrata *relative to *A. thaliana *may come from combining data from relatively more diverged populations (as measured by Φ_*PT*_; Fig. 1) that share little variation. The pooled sample from highly diverged populations would thus consist of predominantly low frequency variants.

To address this issue, we estimated *N*_*e*_*s *separately for each of the populations. The estimates for each *A. lyrata *population were slightly greater than zero in all populations except Germany (Additional file 1: Table S1). In contrast, the *A. thaliana *per-population estimates are slightly negative for two population samples (IN, USA, -0.044; UK, -0.702), slightly positive for a third (NY, USA, 0.169), and undefined (not estimable) for the fourth population (Germany; Additional file 1: Table S1). In this context, it is also important to remember that the models used to estimate *N*_*e*_*s *values assume constant population sizes, and selection-transposition equilibrium [18,33]. As noted previously [5], positive estimates may be misleading because they reflect demographic forces (presumably population bottlenecks during colonization) in the history of individual populations more than selective strength. Many of these assumptions are probably not valid for *A. lyrata *populations [47], but the impact of these assumptions on *A. thaliana *data are less clear. If, for example, *A. thaliana *follows particular kinds of metapopulation dynamics [67], then the approach may be reasonable.

These considerations make it difficult to determine whether there really is a systematic difference in SFS between the outcrosser and the inbreeder. However, *A. lyrata *does trend toward lower average allele frequencies. For example, averaging *p*_*TE *_within populations and taking a grand average across populations, we find that *A. lyrata *has a grand average *p*_*TE *_of 0.250 (sd ± 0.033) and *A. thaliana *has slightly higher grand average of 0.268 (sd ± 0.030). Similar trends are produced by taking the average of medians across populations: *A. lyrata*: 0.189 ± 0.050; *A. thaliana*: 0.198 ± 0.048. Thus, in considering individual populations - as opposed to 'species-wide' samples - there is a slight trend toward lower population frequencies of TEs in *A. lyrata*, consistent with the notion that selection against TEs is stronger in the outcrosser. However, the effect is much muted relative to the species-wide sample, suggesting that population differentiation and demography contributes to some of the differences between pooled, species-wide samples.

### Copy number estimates

The final way in which we compare TEs among populations and between species is by estimating copy number, *n*_*TE *_(see Methods). If selection is more effective in a heterozygous outcrosser, *n*_*TE *_is predicted to be lower in *A. lyrata *than in *A. thaliana*. Per-population *n*_*TE*_, summed over all six shared TE families (Table 2), were not significantly different between species (Wilcoxon rank sum test, *p *= 0.649). Among *A. thaliana *TE families, median *n*_*TE *_varied per population, but *n*_*TE *_varied among populations more in *A. lyrata *(Table 2), perhaps again reflecting relatively higher divergence among *A. lyrata *populations (Fig. 1). In *A lyrata*, as previously noted [5], there is a trend towards lower *n*_*TE *_for each TE family in the German population *vs*. Russia, Sweden, and North America, but no similar clear pattern emerged in the *A. thaliana *data (Table 2).

**Table 2:** Median per individual TE copy number (*n*_*TE*_)

	*A. lyrata*						*A. thaliana*					
	Plech, Germany	North America	Russia	Sweden	Mean	Standard Deviation	Anholt, Germany	IN, USA	NY, USA	Ascot, UK	Mean	Standard Deviation
*Ac*	17.37	23.89	20.44	26.18	21.97	3.87	13.93	11.95	11.92	11.97	12.44	0.99
*Basho*	-	-	-	-	-	-	29.96	40.72	35.76	33.81	35.06	4.48
CACTA	7.19	7.83	6.67	8.6	7.57	0.83	9.92	10.9	9.85	9.9	10.14	0.51
*Gypsy*	14.85	17.77	18.05	12.83	15.88	2.49	19.96	18.95	15.94	14.94	17.45	2.39
LINE	9.47	14.59	13.89	9.34	11.82	2.81	14.97	12.88	13.86	13.83	13.89	0.85
MITE	12.8	13.76	20.65	28.48	18.92	7.27	9.93	21.85	17.79	17.82	16.85	4.99
SINE	17.1	25.26	17.09	21.94	20.35	3.99	25.94	24.86	27.72	23.87	25.60	1.65
Sum*	78.78	103.1	96.79	107.37	96.51	12.59	94.65	101.39	97.08	92.33	96.36	3.87

* *A. thaliana n*_*TE *_sums exclude *Basho *insertions

We observed higher *Ac *copy numbers in *A. lyrata *than in *A. thaliana *in our population-level samples, as measured by the number of TE loci amplified (*A. thaliana n *= 25 *vs*. *A. lyrata n *= 54) or by *n*_*TE *_(mean per population *n*_*TE *_12.44 *vs*. 21.97; Table 2). These results directly contradict those of Wright et al. [42], who detected more copies of *Ac *in *A. thaliana *than in *A. lyrata*. Although our *Ac *bands were amplified using the primers of Wright et al. [42], the sampling strategies differed markedly between studies. The samples of Wright et al. were "species-wide" but uneven, in that 15 populations were represented by a single individual but four populations were represented by > 6 individuals [42]. We believe the contrasting results between studies highlight the effect that sampling can have on subsequent inferences.

Overall, our study is like previous studies in that we detect apparent allele frequency differences between an outcrossing and an inbreeding species but no systematic differences in *n*_*TE *_[38,40,42]. However, several features of our data must be kept in mind: First, the PCR primers were designed from *A. thaliana *genomic sequence, causing a potential ascertainment bias between species. While this bias should not cause difficulties for frequency estimates - which are conditioned on observing a band at an insertion site - this bias could lead to an underestimate of the number of insertion sites in *A. lyrata*. Thus, extrapolating from allele frequencies (*p*_*TE*_) to copy number (*n*_*TE*_) could lead to a systematic underestimate of copy number in *A. lyrata*. Second, TE display bands represent TE sequence found on a limited range of band sizes (~50 to 1000 bases); if there are general differences in TE sizes between species then copy number comparisons may be inaccurate. There is reason to believe that this would trend toward an underestimate in *A. lyrata*: for example, early comparisons of gene structure between the congeners suggest that introns are generally larger in *A. lyrata *[68] and may contribute to the 1.5-fold difference in genome size between the two species [69]. Finally, it is very important to remember that the TE-display protocol amplifies TEs that represent clades or subfamilies of TE families and not entire families. For example, in *A. thaliana *we amplified 52 different MITE elements from the *Tourist*-like subfamily, whereas at least 818 MITEs have been found in the *A. thaliana *genome sequence [9].

## Conclusions

The motivation for this study was to determine whether observed differences in the frequency and population dynamics of TEs can be attributed to species-wide effects, which presumably reflect differences between outcrossing and selfing mating systems, or are better attributed to factors like transposition dynamics and demographic history that may also differ between species. Our study is unique in that we sampled multiple TE families and multiple populations to compare population dynamics between a selfer (*A. thaliana*) and an outcrosser (*A. lyrata*).

Our results indicate that patterns of genetic diversity are heterogeneous across two of the seven surveyed TE families. Unlike other elements, *Bashos *were amplifiable within *A. thaliana *but apparently absent from *A. lyrata*. These observations are consistent with molecular evolutionary analyses that suggest recent bursts of *Basho *insertions within *A. thaliana *[70] and an apparent lack of some *Basho *subfamilies from *A. lyrata *[54]. *Ac *element diversity also differed substantially from other element families, exhibiting low levels of TE band diversity (Fig. 1) and statistically low values of Φ_*PT *_(Fig. 2) within *A. thaliana*. These *Ac *observations could be consistent either with a lack of recent transposition or particularly strong selection targeting new insertions. In any case, our *Basho *and *Ac *results clearly demonstrate that TE dynamics can vary between species and among TEs. They also underline the importance of sampling multiple TE families to make robust inferences about TE dynamics. Although few analyses of TE have studied more than one TE family (albeit in a single species and ignoring between population variation, e.g., [18]), virtually all previous population genetic analyses of TE diversity and mating systems have analyzed data from a single TE family and generalized about mating system dynamics from this single observation [38,40,42]. Such generalizations inherently assume that a single TE family represents the TE complement within a genome, and this may be a poor assumption.

Our data also clearly demonstrate that demographic history shapes TE diversity, because pairwise comparisons involving geographically closer populations often have lower Φ_*PT *_values (Fig. 1). In *A. lyrata*, demographic events perturb selection-transposition equilibria and influence the distribution and frequency of TEs [5,33,47]. Presumably demographic events play a similar role in *A. thaliana*, although the magnitude of these effects is difficult to estimate with the present data.

Finally, some aspects of the data cannot be easily attributed to demography or transposition and thus may reflect differences due to host mating system. These include: the apportionment of diversity within and between populations (Fig. 3); an SFS that provides a consistently higher signal of higher TE population frequencies in *A. thaliana *at both species-wide (Fig. 4) and population scales; and apparent differences in selection coefficients (Fig. 5). Generally, our results contribute to a growing empirical literature that suggests reduced efficacy of selection against TE insertions within selfing lineages [38,40,42], but many questions remain to be addressed about the generality of this observation across taxa and the relative importance of the mechanisms (e.g., ectopic recombination, reduced population sizes, lower effective recombination rates) that may contribute to this effect.

## Authors' contributions

SL carried out the molecular biology work, analyzed the data, and drafted the manuscript. BSG designed the study, assisted with data analysis, and helped draft the manuscript. Both authors read and approved the final manuscript.

## Supplementary Material

Additional file 1 ***N*_*e*_*s *maximum likelihood estimates**. Maximum likelihood estimates of the strength of selection, including 95% confidence intervals, for TEs pooled across populations and across TE families in both species. Click here for file
